# Chitosan particle stabilized Pickering emulsion/interleukin-12 adjuvant system for Pgp3 subunit vaccine elicits immune protection against genital chlamydial infection in mice

**DOI:** 10.3389/fimmu.2022.989620

**Published:** 2022-11-23

**Authors:** Mingyi Shu, Lanhua Zhao, Keliang Shi, Wenbo Lei, Yewei Yang, Zhongyu Li

**Affiliations:** Institute of Pathogenic Biology, Hunan Provincial Key Laboratory for Special Pathogens Prevention and Control- Hengyang Medical School, University of South China, Hengyang, China

**Keywords:** chlamydia trachomatis, Pgp3 protein, chitosan particle stabilized Pickering emulsion, interleukin (IL)-12, immune protection

## Abstract

Considering the shortcomings in current chlamydia infection control strategies, a major challenge in curtailing infection is the implementation of an effective vaccine. The immune response induced by *C. trachomatis* plasmid encoded Pgp3 was insufficient against *C. trachomatis* infection, which requires adjuvant applications to achieve the robust immune response induced by Pgp3. There is increasing promising in developing adjuvant systems relying on the delivery potential of Pickering emulsions and the immunomodulatory effects of interleukin (IL)-12. Here, owing to the polycationic nature, chitosan particles tended to absorb on the oil/water interphase to prepare the optimized chitosan particle-stabilized Pickering emulsion (CSPE), which was designed as a delivery system for Pgp3 protein and IL-12. Our results showed that the average droplets size of CSPE was 789.47 ± 44.26 nm after a series of optimizations and about 90% antigens may be absorbed by CSPE owing to the positively charged surface (33.2 ± 3mV), and CSPE promoted FITC-BSA proteins uptake by macrophages. Furthermore, as demonstrated by Pgp3-specific antibody production and cytokine secretion, CSPE/IL-12 system enhanced significantly higher levels of Pgp3-specific IgG, IgG1, IgG2a, sIgA and significant cytokines secretion of IFN-γ, IL-2, TNF-α, IL-4. Similarly, vaginal chlamydial shedding and hydrosalpinx pathologies were markedly reduced in mice immunized with Pgp3/CSPE/IL-12. Collectively, vaccination with Pgp3/CSPE/IL-12 regimen elicited robust cellular and humoral immune response in mice resulting in an obvious reduction of live chlamydia load in the vaginal and inflammatory pathologies in the oviduct, which further propells the development of vaccines against *C. trachomatis* infection.

## Introduction

Over an estimated 100 million people acquired infection worldwide with *C. trachomatis* each year, a gram-negative obligate intracellular bacterium, leading to a serious public health burden globally because of causative disease sequelae and complications (1). *C. trachomatis* is divided into three biovars and nineteen serovars, which can cause human diseases by infecting eyes, genital tract, gastrointestinal tract, and respiratory tract (2, 3). For example, serovars D~K (the genital tract biovar) are highly prevalent bacterial sexually transmitted infection agents resulting in pelvic inflammatory disease, infertility, ectopic pregnancy, even newborns conjunctivitis, and pneumonia. Serovars A–C (the trachoma biovar) are the leading cause of acquired blindness, whereas other serovars L1–L3 cause the disease lymphogranuloma venereum. *C. trachomatis* infections are often asymptomatic resulting in recurrent infections occurrence. Considering the shortcoming in the diagnosis and treatment of asymptomatic patients and no cost-effective antibiotic drugs for eliminating the pathogen (4, 5), it is a priority to develop an effective vaccine against *C. trachomatis*.

Although a great deal of evidence from animal models or human trials provided by investigators demonstrated that vaccines elicited protection against challenge, human vaccines existing for *C. trachomatis* are not licensed currently. Initial trials of inactivated organism vaccines in humans and non-human primates focused on ocular infection for trachoma prevention (6). However, the duration of immune protection was relatively short and there were security risks owing to the large-scale replication and virulence recovery of inactivated vaccines (6), which facilitated a shift away from whole bacteria vaccination to subunit vaccination strategies. Subunit vaccines are prepared from the main protective immunogen components of *C. trachomatis*, which are capable of motivating immune response against infections. Such as the major outer membrane protein (MOMP), the chlamydial protease-like activity factor (CPAF), heat shock protein 60 (HSP60), the polymorphic membrane proteins (Pmps) and the plasmid-encoded protein (Pgp3) (7–13), which have been tested as subunit vaccine against genital and respiratory challenges in mouse models. Pgp3, the only plasmid encoded protein secreted into the cytoplasm of infected host cells, is a key virulence factor and immunodominant protein required for colonization, persistent infection establishment and associated immunopathology of *C. trachomatis* (14–16). In previous studies, Li et al. have certified that Pgp3 was immunogenic protein with higher titer by reacting with antibodies from women urogenitally infected with *C. trachomatis* (17). In addition, the immune response level and protective immunity was demonstrated by immunization with Pgp3 DNA vaccine (18, 19). Thus, there was the promising vaccine candidate relying on Pgp3 as recombinant subunit vaccine. However, the protein vaccines usually have poor immunogenicity resulting in failing to achieve high-efficient and long-term protective immunity. Therefore, it is necessary that develops adjuvant platforms for the vaccines to induce robust immune responses.

Actually, an adjuvant platform containing an antigen delivery system and an immunostimulant has presumably been recognized as an effective strategy. The best delivery system for protein antigens can enhance antigens uptake by antigen-presenting cells (APCs) and prolong the residence time of injected antigens, while the immunopotentiator activates innate immunity by providing the immune molecular signals (20, 21). Recent studies have suggested that enhancement of antigens uptake by APCs relying on the potential of Pickering emulsions contributed to both humoral and cellular responses stimulation (22, 23). As emulsions are stabilized by solid particles, Pickering emulsions have attracted increased research attention owing to high biosafety, stability, and antigen-loading capability (24). In addition, there are considerable research advancements in the encapsulation and delivery of bioactive compounds, skin applications, environmental applications, microcapsules, and highly porous materials because of biodegradable, biocompatible, and nontoxic properties exist for CSPE (25–28). The safe, stable, and effective delivery characterization of CSPE may make it a strong adjuvant candidate for subunit vaccines of *C. trachomatis* even if no research as antigens delivery systems.

Interleukin (IL)-12 remains a very promising immune booster because of cellular immunity induction by stimulating T-cell proliferation and natural killer (NK) cells activation to secret interferon (IFN)-γ (29), which has been widely tested as an adjuvant due to potentially shift immune response from a Th2 to a Th1 (30–35). As demonstrated by animal models, some research has shown that extending the persistent time of injected IL-12 may improve its auxiliary efficacy. For instance, vaccination of protein vaccine plus persistent IL-12 effect produced higher and longer immunity protection against leishmania infections, suggesting durable cell-mediated immune responses may be maintained by the persistence of IL-12 (36). Similarly, more effective immunity appeared in protein vaccines adjuvanted with the combination of IL-12 and aluminum hydroxide (alum), this is because the residence time of IL-12 was prolonged by alum leading to the persistence of immune enhancement effects (37, 38). Therefore, we hypothesized that the persistence of IL-12 may be extended at the injection site by delivery systems, which may be contributed to its adjuvanticity enhancement.

Based on the delivery potential of CSPE for soluble proteins and immunoenhancement effects of IL-12, we proposed a hypothesis that an effective adjuvant system for intraperitoneally administering Pgp3 protein was vaccinated by combining CSPE with IL-12. Here, we conducted a series of optimizations to prepare CSPE and subsequently combined it with recombinant mouse IL-12 to formulate CSPE/IL-12 system for adjuvant application. The efficacy of the CSPE/IL-12 adjuvant system for Pgp3 protein *in vivo* was evaluated using mice models infected by *Chlamydia muridarum*. Our results showed that humoral and cellular immune response induced by Pgp3 protein were greatly enhanced by CSPE/IL-12 adjuvant system, as characterized by high levels of Pgp3-specific antibodies (IgG, IgG1, IgG2a, sIgA) and cytokines production (IFN-γ, IL-2, TNF-α, IL-4) following vaccination regimen. Given robust immune response induction, vaccination with Pgp3/CSPE/IL-12 resulted in a reduced genital chlamydial load in the lower genital tract and less inflammation pathology in the upper reproductive tract, which may provide insights for the development of an efficient and safe adjuvant strategy for *C. trachomatis* vaccine.

## Materials and methods

### CSPE preparation

Chitosan solutions were prepared by dissolving the 15 kDa molecular weight of dry powder (Golden-Shell Biochemical Inc, Zhejiang, China) in deionized water and an appropriate amount of acetic acid was simultaneously added to the solutions. Then the solutions were mixed with a magnetic stirrer (Thunder Magnetic, Shanghai, China) overnight at a rate of 220 rpm/min and at room temperature. After the impurities in the resulting chitosan solutions were filtered *via* G3 sand core funnel, the pH of chitosan solutions was adjusted using NaOH (1M) or HCL solutions (1M) to form chitosan nanoparticles. CSPE was prepared by mixing chitosan nanoparticles and liquid paraffin at a certain solutions-oil volume ratio and subsequently ultrasonically homogenizing the mixture by an ultrasonic homogenizer, which power was 110-440 W and time was 4 min with 4 s pulse on, 4 s pulse off.

### CSPE characterization

The droplet shape of emulsions was observed with an optical microscope (Nikon Corporation, Japan). The droplet size and zeta potential of emulsions were measured by Zeta sizer Nano-ZS analyzer (Malvern Instruments, UK) at 25°C after the samples were diluted 100 times.

### Growth of *C. muridarum* stocks

HeLa cells were maintained in DMEM medium (HyClone, South Logan, UT, USA) containing 10% FBS at 37°C, 5% CO_2_ condition, and infected with *C. muridarum* using standard procedures as described previously (39). EBs were purified from Hela cells infected with *C. muridarum* and stored in sugar phosphate glutamate buffer (SPG) at −80°C. The stock of *C. muridarum* inclusion forming units (IFU) was tittered in Hela cells.

### Fluorescence assay

Rhodamine (Sinopharm Chemical Reagent Co, Shanghai, China) solution dissolved in DMSO (1mg/mL) was taken to the chitosan solutions and then the mixture solution was shaken for 4-10h. The dialysis bag (Solarbio, Beijing, China) was employed to remove unbound rhodamine dye in the mixture solution. Chitosan solution combined with rhodamine dye was adjusted to the appropriate pH to prepare Pickering emulsion after oil addition. FITC-BSA (Solarbio, Beijing, China) was mixed with CSPE on ice for 4h according to vaccine regimens, and subsequently, the CSPE/FITC-BSA mixture was co-cultured with macrophages for 2h at a 37°C, 5% CO2 incubator. After culturation and washing by PBS, the macrophages were fixed with 4% paraformaldehyde (PF, Sigma-Aldrich, St. Louis, Missouri, USA) at 4°C for 30min. After being permeabilized by 0.5% TritonX-100 (Sigma-Aldrich, St. Louis, Missouri, USA) and blocked by RPMI-1640 containing 10% FBS, macrophages were incubated with YF^®^594-Phalloidin (US EVERBRIGHT, Silicon Valley, California, USA) at room temperature for 2h, DAPI (Sigma-Aldrich, St. Louis, Missouri, USA) was added to visualize the nuclear DNA. Images of the above samples were observed with a fluorescence microscope (Nikon, TE2000-S, Japan).

### Animal ethics statement

4-weeks old female BALB/c mice were obtained from Hunan SJA Laboratory Animal Co. Ltd (Animal Production License No. SYXK 2021-0002) and kept in the Animal Experiment Research Center at the University of South China. All experimental protocols involving animals were approved by the Ethics Committee of the Animal Experiment Research Center at the University of South China. The mice operations conformed to National Laboratory Animal Care and Use Guidelines.

### Mice immunization

Pgp3 protein was extracted using standard procedures described previously (40) and CSPE was mixed with recombinant murine IL-12 to prepare the adjuvant. Each mouse was intraperitoneally immunized three times at two-week intervals with 100μL vaccine prepared by mixing 50μg Pgp3 protein and adjuvant. All groups included Pgp3 alone, Pgp3/CSPE, Pgp3/IL-12, Pgp3/CSPE/IL-12 and negative control group PBS. Mice serum samples were collected the day before per vaccination and stored at -20°C. Two weeks after the final immunization, serum samples and vaginal washes liquid were harvested and stored at -20°C to determine specific antibody production. Meanwhile, spleens were collected by filtering through a 70-mm nylon mesh (BD Falcon, New York, USA) and treatment with RBC lysis buffer (Invitrogen, California, USA).

### Antibody titer detection

Pgp3-specific antibody titers were measured by ELISA (7, 41). Briefly, after being coated overnight at 4°C with 10μg/well of Pgp3 in 100µL coating buffer solution, 96-well plates (JET BIOFIL, Guangzhou, China) were washed using PBS solution containing 0.05% Tween 20 (PBST) and blocked with 250μL PBST plus 5%BSA for 2h at 37°C. 100μL serially diluted serum samples and 100μL vaginal washes liquid were added to each well following washing. After incubation for 2 h at 37°C, the plates were washed and then incubated with 100μL/well of a 1:5000 dilution of horseradish peroxidase (HRP)-conjugated goat anti-mouse IgG, IgG1, IgG2a, IgA (Proteintech, Chicago, USA) at 37°C for 1 h, respectively. Washing again and the binding was quantified by adding 100μL/well TMB substrate at 37°C for 15min. The reaction was stopped by 50μL/well H_2_SO_4_ and the absorbance of each well at 450 nm was measured by an EIA reader (Bio-Rad, Hercules, CA, USA).

### Detection of cellular immune response

Lymphocyte proliferation assay and antigen-specific cytokine analysis were performed using splenocytes. The harvested splenocytes were resuspended with RPMI-1640 medium containing 10%FBS and cultured in 24-well plates or 96-well plates at the right density. The splenocytes were stimulated with 10μg/well Pgp3 or 5μg/mL positive control ConA (Invitrogen, California, USA) for 48h and then were measured using a Cell Counting Kit-8 (CCK8, DOJINDO Molecular Technologies, Gaithersburg, MD, Japan) for evaluating its proliferative capacity. In addition, the splenocytes were cultured in 24-well plates and stimulated by 10μg/well Pgp3 or medium for 48h. After stimulation, the supernatants were collected to detect IFN-γ, IL-2, TNF-α and IL-4 secreted from splenocytes according to the manufacturer’s instructions (Biolegend, San Diego, California, USA).

### Genital challenge and vaginal chlamydial load

Seven days prior to *C. muridarum* challenge, each mouse was injected with 2.5mg medroxyprogesterone (XIANJU PHARMA, Zhejiang, China) to synchronize the estrus cycle, and then was intravaginally challenged with 2×10^5^ IFU of *C. muridarum* in 15μL SPG at 30 days after the last immunization. The vaginal samples were collected with swabs at 3- or 4- days intervals until shedding was negatively detected for two consecutive time points. The vaginal chlamydia load was evaluated and counted by fluorescent staining as described (42–44).

### Evaluation of mice genital tract pathology

Mice were sacrificed 60 days after challenge to evaluate tissue pathology by harvesting entire genital tracts including the vagina, the uterus, the oviduct, and the ovary. Gross examination *in situ* was necessarily performed to observe oviduct hydrosalpinx or any other related abnormalities before removing the genital tract tissues, and then the genital tract tissues were isolated entirely and put on a blue background for the acquisition of images. Visual scores of the oviduct hydrosalpinx were assessed based on dilation size under a scoring system (44): no oviduct dilation (0); hydrosalpinx existence under a stereoscope but not with naked eyes (1); The diameter of visible hydrosalpinx with naked eyes less than ovary (2); The diameter of hydrosalpinx is equal to the ovary (3); The diameter of hydrosalpinx is larger than ovary (4). For scoring of histological damage and inflammatory cells infiltration, paraffin sections of the isolated tissues were prepared and stained with hematoxylin and eosin (H&E) according to reference methods (14). The histological scoring and inflammatory cell counting were referred to with the established scoring system as in the previous article (45). Dilation was scored by the following criteria: no obvious dilation (0); one slightly dilated transverse section is visible (1); two to three dilated cross sections (2); more than three dilated cross sections (3); confluent significant dilation (4). Inflammatory infiltration scoring: no infiltration (0); one infiltration foci (1); two to four infiltration foci (2); more than four infiltration foci (3); confluent infiltration (4).

### Statistics analyses

The Student’s t-test and one-way analysis of variance (ANOVA) were used for statistical analysis, GraphPad Prism 8 software was employed for data processing, analysis, and graphical representation. *P*<0.05 was accepted as statistically significant.

## Results

### Characterization of CSPE

**Figure 1:**
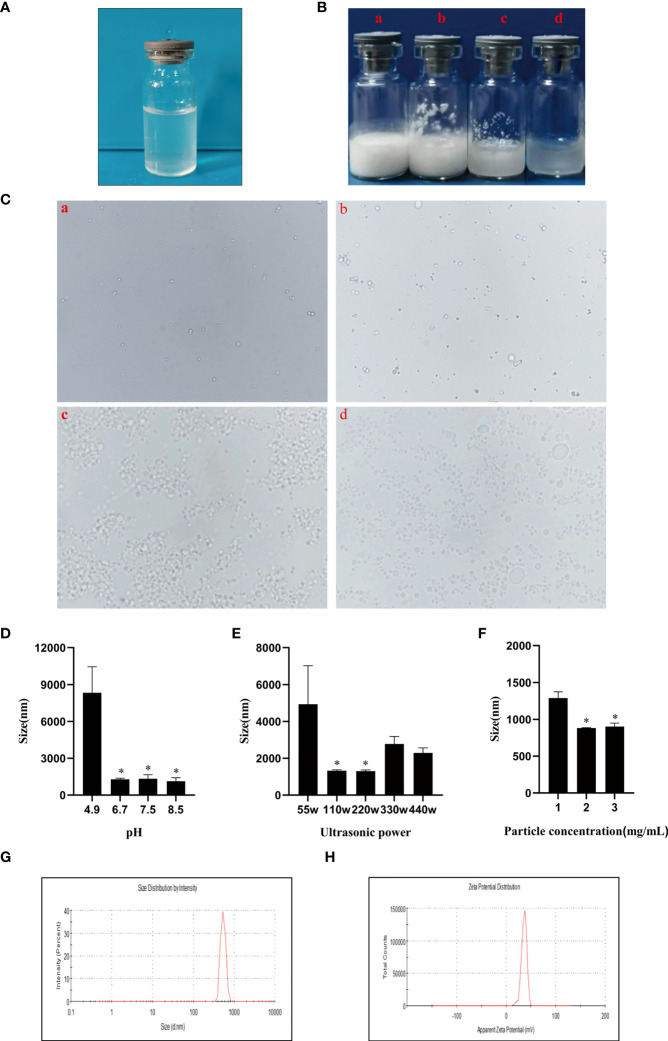
CSPE preparation and characterization. **(A)** The appearance of chitosan solution after its pH was adjusted to the appropriate value. CSPE was prepared through mixing chitosan nanoparticles and oil phase, and subsequently ultrasonically homogenizing the mixture under an ultrasonic homogenizer. **(B)** The appearance of CSPE prepared by mixing chitosan particles and oil phases such as liquid paraffin **(a)**, squalene **(b)**, soybean oil **(c)** and n-hexane **(d)**. **(C)** Microscopic image of CSPE droplets by prepared by mixing chitosan particles and oil phases such as liquid paraffin **(a)**, squalene **(b)**, soybean oil **(c)** and n-hexane **(d)**. **(D)** The average size of CSPE droplets prepared at different pH of chitosan solution. **(E)** The average size of CSPE droplets prepared under various ultrasonic power. **(F)** The average size of CSPE droplets prepared at different chitosan particles concentration. The most optimized CSPE was prepared through mixing liquid paraffin and 2mg/mL chitosan particles following adjustment of pH and subsequently ultrasonically homogenizing the mixture at 110 W ultrasonic power. **(G)** The size distribution of optimized CSPE. **(H)** The zeta potential distribution of optimized CSPE. All data are representative of three independent experiments, *P<0.05.

As a stabilizer of Pickering emulsions, hydrophobic particles tend to absorb on the two-phase interface to block droplets coalescence, thereby conferring emulsions high stability. And the droplet size and stability are important parameters for Pickering emulsions, which may be influenced by factors including ultrasonic power, solution pH, particle concentration, oil phase species, and so on (27). Therefore, we attempted to prepare the most optimized CSPE by utilizing various strategies. After the pH of chitosan solution was adjusted to the appropriate value, the appearance of chitosan solution was shown in **Figure 1A**. **Figure 1B** exhibited the appearance of CSPE prepared by mixing chitosan particles and oil phase species such as liquid paraffin (**Figure 1B.a**), squalene (**Figure 1B.b**), soybean oil (**Figure 1B.c**) and n-hexane (**Figure 1B.d**), and the corresponding microscopic image of CSPE droplets appeared spherical in shape (**Figure 1C**). The results showed that emulsions formed by squalene and soybean oil were not fine fluid because of the sticky substance attached to the bottle, and inadequate preparation of CSPE using n-hexane (**Figure 1B**). Similarly, uneven size of spherical droplets was presented in microscopic images of soybean oil and n-hexane. These results suggested that instability and inhomogeneity of CSPE were prepared by squalene, soybean oil, and n-hexane. On the contrary, there was a delicate fluid state from the appearance and small as well as uniform droplets under the microscope in CSPE formulated by liquid paraffin and chitosan particles (**Figures 1B.a**, **1C.a**), indicating that liquid paraffin was the suitable candidate on oil phase selection.

Chitosan is soluble in dilute acid aqueous solution owing to the protonation effect of polycationic properties. The free amino groups of chitosan dissolved in dilute acid aqueous solution are largely protonated leading to the hydrophilicity increase, which is not suitable as a particle emulsifier. However, at a high pH, deprotonation of chitosan will cause the hydrophobicity to augment the intermolecular attraction, which consequently forms particles suitable for Pickering emulsion preparation by undergoing interpolymer associations (27, 28). After liquid paraffin addition, it was found that the droplet size of CSPE was significantly decreased when pH of the solution ≥ 6.7 compared with solutions pH at 4.9, this may be because hydrophobic chitosan particles required for stabilizing Pickering emulsions were associated with the protonation of free amino groups in chitosan (**Figure 1D**). In addition, as shown in **Figure 1E**, the size of spherical droplets was influenced by ultrasonic power because the emulsion was not effectively sheared leading to a larger size at low ultrasonic power and poor stability at excessive ultrasound power. Therefore, the most optimized CSPE was prepared at the appropriate ultrasonic power.

Pang B et al. have demonstrated that droplet size decreased with increasing the stabilizer concentration (24, 27), **Figure 1F** revealed this connection between droplet size of CSPE and chitosan concentration. However, the particle size of the emulsion didn’t continuously decrease at a chitosan concentration of 3mg/mL, indicating that there were enough particles to stabilize the emulsion at a chitosan concentration of 2 mg/mL. Collectively, the most optimized CSPE was prepared by mixing liquid paraffin and 2 mg/mL chitosan particles following adjustment of pH, and subsequently ultrasonically homogenizing the mixture at 110 W ultrasonic power. **Table 1** revealed the average size, average Zeta potential, and PDI of the most optimized CSPE. Correspondingly, the size distribution and zeta potential distribution were respectively shown in **Figures 1G**, **1H**, demonstrating excellent stability and fine homogeneity existed in CSPE.

**Table 1:** Characterization of CSPE.

Characterization	
Size	789.47±44.26nm
Zeta potential	33.2±3mV
PDI	0.427±0.12
Antigen absorption efficiency (100%)	89.04±3.8

### Location of chitosan particles in CSPE and antigen adsorption by CSPE

**Figure 2:**
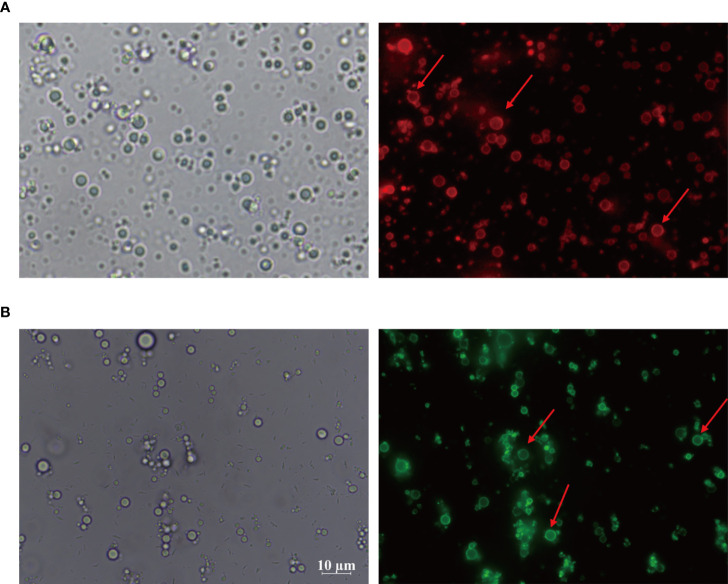
Rhodamine was taken to the chitosan solutions and then the mixture solution was shaking for 4-10 h to obtain rhodamine labeled chitosan particles. CSPE prepared by rhodamine labeled chitosan particles and liquid paraffin. **(A)** Microscopic image and corresponding fluorescence image of CSPE. FITC-BSA was served as model antigen and was mixed with CSPE on ice for 4h. **(B)** Microscopic image and corresponding fluorescence image of CSPE.

In order to investigate the distribution of chitosan particles in emulsions droplets, chitosan particles combined with rhodamine dye were applicated to produce Pickering emulsion after oil addition and the microstructure of the emulsion droplets was analyzed by fluorescence microscope. **Figure 2A** revealed that chitosan particles tended to adsorb at the droplet interface to stabilize the emulsion droplets, which was consistent with the stabilization mechanism of Pickering emulsions. Moreover, there are generally two strategies to load antigens including embedding and adsorption (46–48), FITC-BSA was used as a model antigen for loading analysis. After co-incubation of CSPE and FITC-BSA, we demonstrated that CSPE adsorbed FITC-BSA according to the fluorescence image in **Figure 2B**, indicating that CSPE may load protein antigen by adsorption. Next, we mixed the most optimized CSPE and Pgp3 protein according to vaccine regimens for adsorption rate evaluation. As shown in **Table 1**, about 90% of Pgp3 protein may be adsorbed by CSPE within 4h.

### CSPE enhances antigen uptake by macrophages

**Figure 3:**
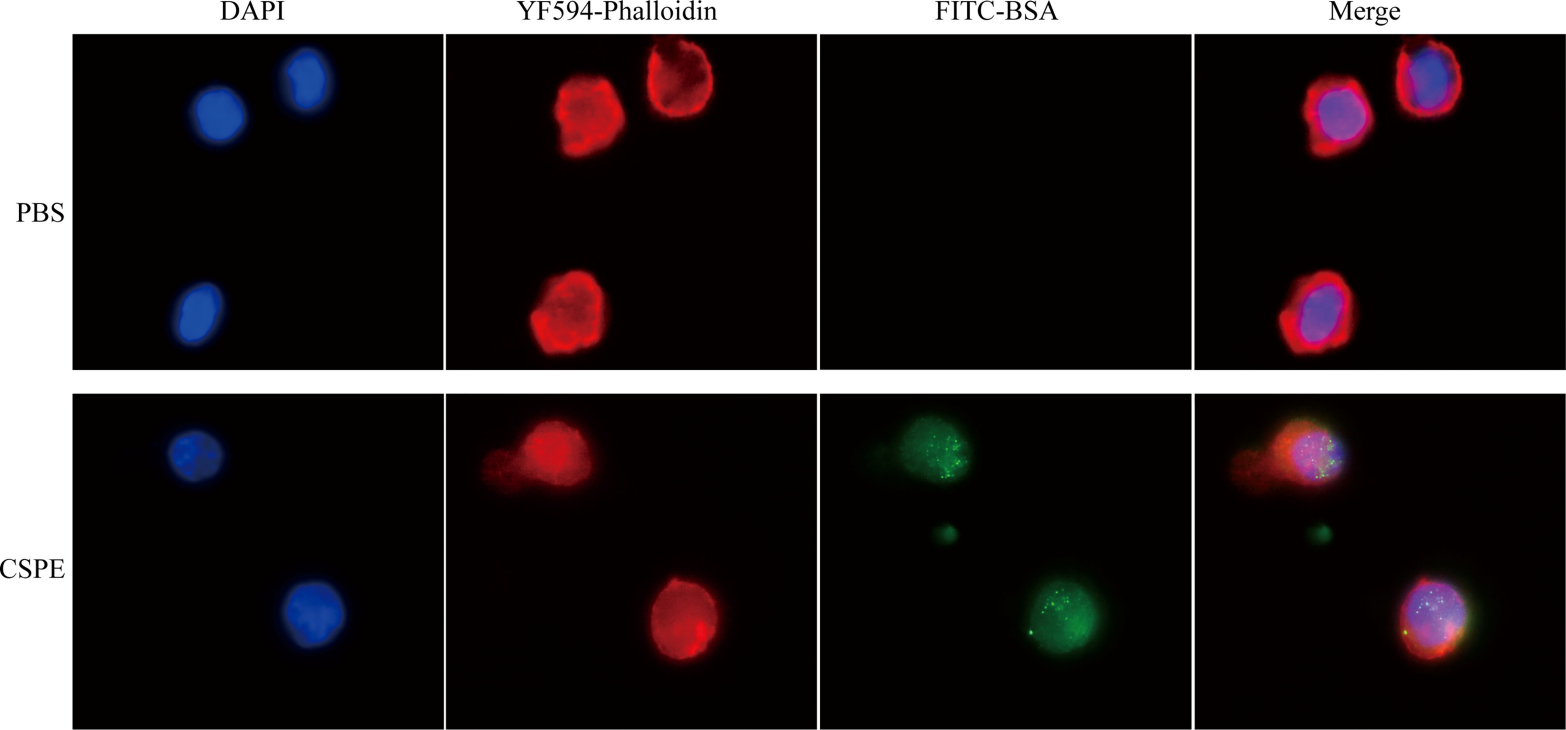
FITC-BSA was mixed with CSPE on ice according to vaccine regimens and the mixture was co-cultured with macrophages for 2h at a 37°C, 5% CO_2_ incubator. The phagocytosis process of CSPE/FITC-BSA complexes by macrophages. Membrane actin and nuclear DNA were labelled by YF^®^594-Phalloidin (red) and DAPI (blue), respectively.

Considering the importance of sensation and internalization of antigens by APCs for cellular immunity activation, the Pickering emulsions may be contributed to enhancing antigens uptake by APCs and subsequent APCs activation depending on its delivery potential (22, 23, 49). As multifunctional phagocytic cells, macrophages are allowed to efficiently sense and internalize endogenous and exogenous material, such as viruses, microorganisms, cells, cell debris, and foreign particulate matter. Therefore, we examined the interaction between macrophages and CSPE-absorbed FITC-BSA by fluorescence analysis. The phagocytosis process of CSPE/FITC-BSA complexes by macrophages was shown in **Figure 3**, DAPI and YF594-Phalloidin were respectively stained nucleus-DNA and membrane actin. Consequently, CSPE/FITC-BSA complexes were found in macrophages compared with PBS group, suggesting that CSPE may have the potential to enhance antigens uptake by macrophages for strengthening cellular responses. Therefore, it seems promising to develop an efficient adjuvant for Pgp3 protein vaccines owing to potent macrophage-residing CSPE/FITC-BSA complexes.

### Humoral immune response evaluation

**Figure 4:**
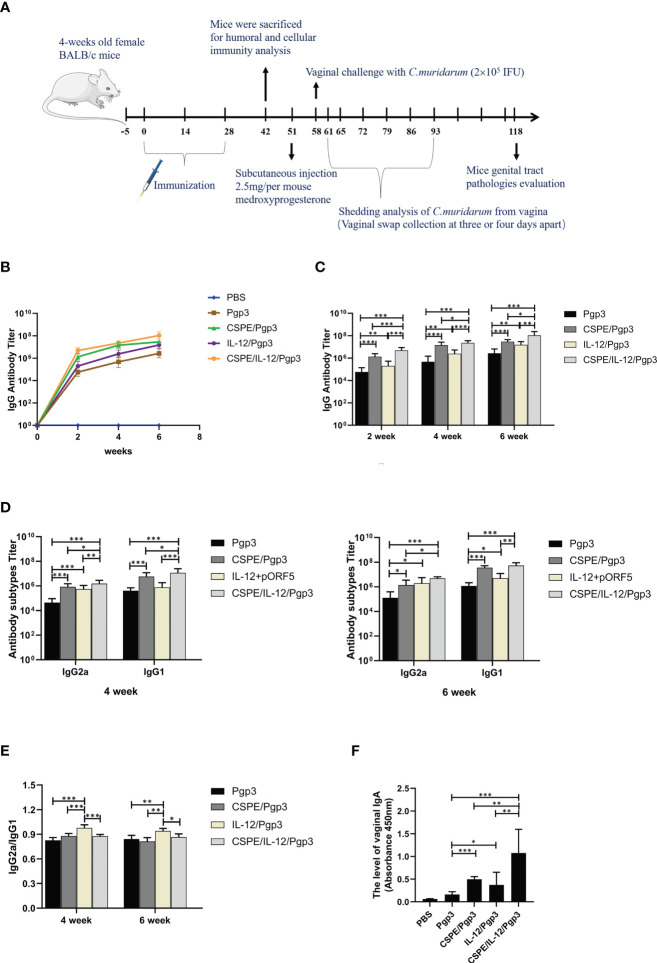
Pgp3-specific humoral immune response. **(A)** Schematic of the experimental design for the immunization timeline and vivo work in mice. Five groups of BALB/c mice (n=10 in each group) were immunized three times at two-week intervals with Pgp3/CSPE, Pgp3/IL-12, Pgp3/CSPE/IL-12, Pgp3 alone and PBS (mock control). Serum samples were collected the day before per vaccination and two weeks after the final immunization from immunized mice to determine Pgp3-specific antibody levels by ELISA. **(B)** Trend of IgG levels after immunization in mice of each group. **(C)** IgG levels in each group after immunization. **(D)** IgG2a and IgG1 levels in each group after immunization. **(E)** IgG2a/IgG1 ratio in each group. Two weeks after the final immunization, vaginal washes liquid was harvested to determine specific sIgA production. **(F)** The level of vaginal IgA in each group. The data are representative of three independent experiments, *P<0.05, **P<0.01, ***P<0.001.

A schematic of the experimental design for the immunization timeline and *in vivo* work in mice was shown in **Figure 4A**. Specific serum antibodies (total IgG, subtypes G1 and G2a) and secretory IgA (sIgA) play vital effects in protective humoral immune response and mucosal immune responses against pathogen infections (50). Apparently, the results revealed that the IgG level continuously raised with increasing immunization times in **Figure 4B**, and significantly higher levels of Pgp3 specific IgG, IgG1, IgG2a, and sIgA were detected by ELISA in Pgp3/CSPE, Pgp3/IL-12 and Pgp3/CSPE/IL-12 vaccination than Pgp3 vaccination, indicating encouragement on humoral immunity by the adjuvant activity. Two weeks after the last immunization, higher IgG titer was induced by CSPE/IL-12 adjuvant system compared with Pgp3/CSPE group and Pgp3/IL-12 group (**Figure 4C**, *P*<0.05). In addition, compared with Pgp3/CSPE/IL-12 group, it was found that about two times reduction of IgG2a and IgG1 levels were detected in Pgp3/CSPE group in the fourth week, and the levels of IgG2a and IgG1 induced by IL-12 adjuvant were decreased by 102% and 1420%, respectively (**Figure 4D**, *P*<0.05). Obviously, these results showed that CSPE/IL-12 maintained a high level of IgG2a and IgG1 than other groups. Of note, the more presence of IgG2a and IgG2a/IgG1 ratio may be indicative of a Th1-polarized immune (51, 52). As indicated in **Figure 4E**, IgG2a/IgG1 ratios in Pgp3/IL-12 group were significantly elevated while IgG1 production was lower compared with other groups, which indicated a mixed Th1/Th2 response induced by IL-12 with a bias toward Th1. However, CSPE and CSPE/IL-12 tended to promote mixed Th1 and Th2 immune responses because of high IgG1 and IgG2a levels. Next, the sIgA levels in vaginal secretions were detected to compare the mucosal responses. After absorbance value analysis, **Figure 4F** revealed that more than two times higher sIgA secretion induced CSPE/IL-12 than CSPE or IL-12 treated groups (*P*<0.05). Taken together, these results demonstrated that the mixed Th1 and Th2 immune response induced by Pgp3/CSPE/IL-12, which indicated that the combination of CSPE and IL-12 was conducive to the elevation of the robust humoral immune response.

### Specific splenocytes cell response assessment

**Figure 5:**
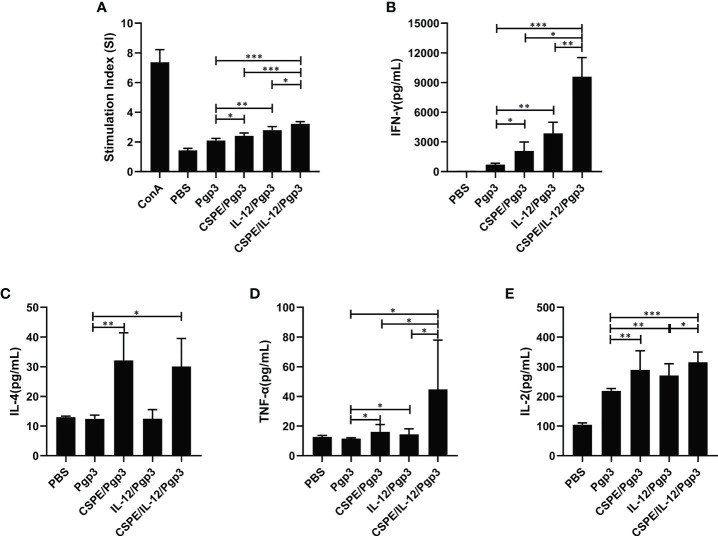
Pgp3-sepecific cellular immune response. Splenocytes were collected and then stimulated with medium and Pgp3 for evaluating lymphocyte proliferation and antigen-specific cytokine. **(A)** Different immunization groups induced Pgp3-specific proliferative responses in splenocytes. The levels of IFN-γ **(B)**, IL-4 **(C)**, TNF-α **(D)** and IL-2 **(E)** secreted from splenocytes were analyzed according to the manufacturer instructions. The data are representative of three independent experiments, *P<0.05, **P<0.01, ***P<0.001.

To determine the cell-mediated immune response induced by different immune strategies. At day 14 after the last immunization, mice were sacrificed to harvest splenocytes and subsequent splenocytes stimulation using Pgp3 protein. As shown in **Figure 5A**, Pgp3 vaccination plus adjuvant enhanced splenocyte proliferation ability compared with PBS or Pgp3 groups. The immunization with Pgp3/CSPE/IL-12 generated a significantly higher proliferation response than other groups (*P*<0.05). Moreover, several typical splenocyte cytokines secretions such as IFN-γ, IL-2, TNF-α, and IL-4 were detected according to the manufacturers instructions. The results demonstrated that the level of IFN-γ, IL-2, and TNF-α in Pgp3/CSPE, Pgp3/IL-12, and Pgp3/CSPE/IL-12 mice were increased compared with Pgp3 immunized group. About fivefold higher IFN-γ levels (**Figure 5B**, *P*<0.05) and threefold higher TNF-α levels (**Figure 5D**, *P*<0.05) were induced by CSPE/IL-12 system than that in the CSPE group. The IFN-γ and TNF-α cytokines showed similar tendencies when compared with the IL-12 group. Concurrently, it was found that 17% higher IL-2 (**Figure 5E**, *P*<0.05) were secreted in Pgp3/CSPE/IL-12 mice compared with Pgp3/IL-12 immunized mice. These results suggested that Pgp3/CSPE/IL-12 immunization elicits the cell-mediated immune response, indicating the effectiveness of combined application between IL-12 and CSPE for a cellular response. In addition, the IL-4 production (Th2) was also significantly increased (**Figure 5C**, *P*<0.05) in Pgp3/CSPE/IL-12 groups compared with Pgp3 vaccination. However, IL-12 failed to induce IL-4 secretion, which was consistent with its biological functions (29). Therefore, there was no difference in the IL-4 secretion between Pgp3/CSPE and Pgp3/CSPE/IL-12 groups. Taken together, these results further validated a mixed Th1 and Th2 activation by CSPE/IL-12, which may offer comprehensive immune protection during *C. trachomatis* invasion.

### Pgp3 protein with CSPE/IL-12 system protects against vaginal infection

**Figure 6:**
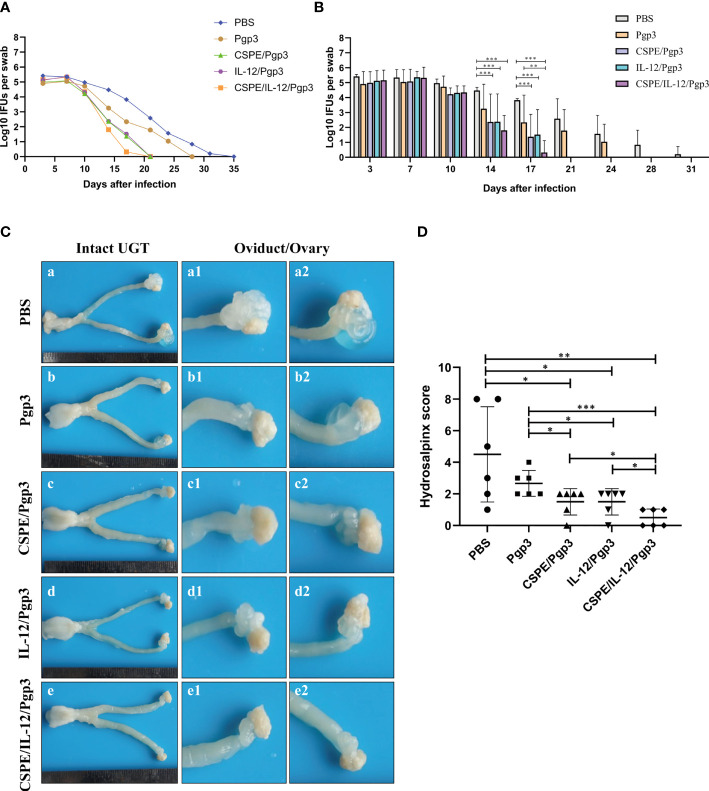
CSPE/IL-12/Pgp3 vaccine formulation reduced chlamydial burden and urogenital tract gross pathology. Animals (six mice/group)were intravaginally challenged with 2×10^5^ IFU of *C. muridarum* on day 58 and then vaginal swabs were harvested to enumerate recoverable IFUs at 3- or 4- days intervals. **(A)** The line chart for chlamydial load of each group was presented in log10 IFUs. **(B)** Corresponding histogram for chlamydial load of each group was presented in log10 IFUs. Mice were sacrificed on the 60 days after challenge to evaluate tissue pathology by harvesting entire genital tracts. **(C)** The representative images of genital tracts gross appearance in mice of PBS **(a)**, Pgp3 **(b)**, Pgp3/CSPE **(c)**, Pgp3/IL-12**(d)** and Pgp3/CSPE/IL-12 **(e)** groups were evaluated under naked eyes. **(D)** Corresponding hydrosalpinx score in each group mice. *P<0.05, **P<0.01, ***P<0.001, vs. analyzed by ANOVA.

To further assess the immune protections, mice were intravaginally challenged with 2×10^5^ IFU of *C. muridarum* at 30 days after the final immunization. Then, the vaginal chlamydia shedding was detected at 3- or 4- days intervals after inoculation to follow the course of the infection (**Tables 2**, **3**, **Figures 6A**, **B**). Overall, there was a consistent decrease in the chlamydial organism shedding of all groups. Pgp3/CSPE, Pgp3/IL-12, and Pgp3/CSPE/IL-12 immunized groups exhibited a notable reduction in the shedding of chlamydia organisms on day 14 and day 17 compared with PBS groups (**Figures 6A**, **B**). The shedding of chlamydia organisms in mice of Pgp3/CSPE/IL-12 immunized group was significantly decreased compared with PBS or Pgp3 groups (**Figure 6B**, *P*<0.05). Similarly, 83.3% of the mice immunized with Pgp3/CSPE/IL-12 resolved infection on day 17 compared with the other groups (**Table 3**). These results demonstrated that vaccination with Pgp3/CSPE/IL-12 accelerated chlamydial clearance in mice genital tract.

**Table 2:** Percentage of mice shedding chlamydia.

Group	%mice Shedding chlamydia from the vagina(n=6)									
	D3	D7	D10	D14	D17	D21	D24	D28	D31	D35
PBS	100	100	100	100	100	83.3	66.7	50	16.7	0
Pgp3	100	100	100	83.3	66.7	66.7	50	0	0	0
CSPE/Pgp3	100	100	100	66.7	50	0	0	0	0	0
IL-12/Pgp3	100	100	100	66.7	50	0	0	0	0	0
CSPE/IL-12/Pgp3	100	100	83.3	83.3	16.7	0	0	0	0	0

**Table 3:** Number of mice that stopped shedding chlamydia with time after challenge.

Group	Number of mice that stopped shedding chlamydia (n=6)									
	D3	D7	D10	D14	D17	D21	D24	D28	D31	D35
PBS	0	0	0	0	0	1	2	3	5	6
Pgp3	0	0	0	1	2	2	3	6		
CSPE/Pgp3	0	0	0	2	3	6				
IL-12/Pgp3	0	0	0	2	3	6				
CSPE/IL-12/Pgp3	0	0	1	1	5	6				

**Figure 7:**
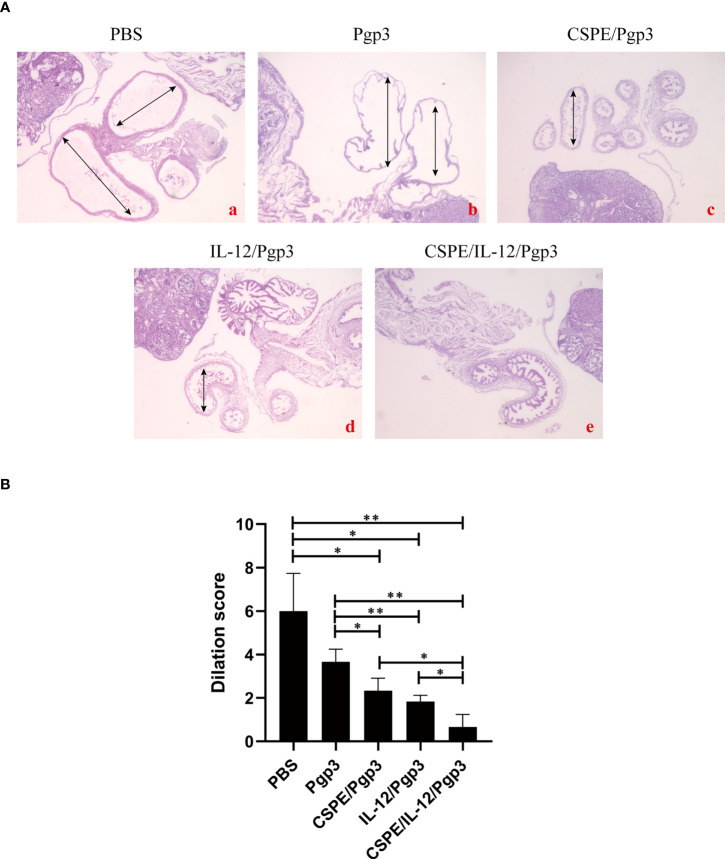
Entire oviduct tissues were harvested from animals (three mice/group) to evaluate oviduct dilation by staining with H&E. **(A)** The representative images from PBS **(a)**, Pgp3 **(b)**, Pgp3/CSPE **(c)**, Pgp3/IL-12 **(d)** and Pgp3/CSPE/IL-12 **(e)** groups were evaluated under microscope. **(a-e)** images were magnified 40×. **(B)** Histograms for dilation scores of each group. *P<0.05, **P<0.01, vs. analyzed by ANOVA.

**Figure 8:**
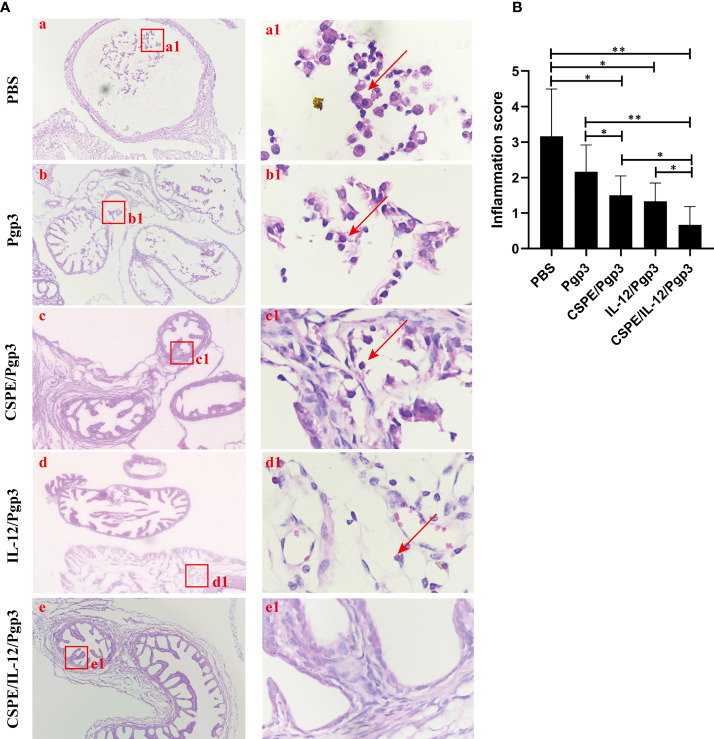
Entire oviduct tissues were harvested from animals (three mice/group) to evaluate inflammatory cells infiltration by staining with H&E. **(A)** The representative images from PBS **(a)**, Pgp3 **(b)**, Pgp3/CSPE **(c)**, Pgp3/IL-12 **(d)** and Pgp3/CSPE/IL-12 **(e)** groups were evaluated under microscope. **(a-e)** images were magnified 400×, red rectangles in the **(a-e)** images indicated the same areas with **(a1-e1)** images. **(B)** Histograms for inflammation scores of each group. *P<0.05, **P<0.01, vs. analyzed by ANOVA.

At day 60 after intravaginal challenge by *C. muridarum*, we evaluated mice pathogenicity by isolating the intact urogenital tract tissues, the representative images of urogenital tract gross pathology and corresponding histopathology in each group were respectively shown in **Figures 6C**, **7A**, and **8A**. Apparently, no obvious dilation in the oviduct tissues of mice vaccinated with Pgp3/CSPE/IL-12 groups, and both severity scores and hydrosalpinx incidence rates were lower than PBS or Pgp3 groups (**Figure 6D** and **Table 4**). Especially in mice immunized Pgp3/CSPE/IL-12, the results showed that unilateral hydrosalpinx existed in 50% of the mice (**Table 4**) and corresponding lower scores of gross hydrosalpinx compared with the other groups (**Figure 6D**), which suggested that Pgp3/CSPE/IL-12 vaccination protected the mice from *C. muridarum* induced hydrosalpinx occurrence. In addition, we assessed oviduct dilation and inflammatory cell infiltration in the oviduct tissue by H&E staining. As expected, *C. muridarum* induced markedly oviduct dilation (**Figure 7B**) and inflammatory cell infiltration (**Figure 8B**) in mice of PBS groups. In contrast, there was a significant reduction of oviduct dilation (**Figure 7B**) and inflammatory cells infiltration (**Figure 8B**) in mice of Pgp3/CSPE, Pgp3/IL-12 and Pgp3/CSPE/IL-12 immunized groups (*P*< 0.05). Contrary to other groups, no significant inflammatory infiltration and dilation was found in mice immunized with Pgp3/CSPE/IL-12. Generally, these results indicated that vaccination with Pgp3/CSPE/IL-12 protected the mice from chlamydia-induced upper genital tract pathology.

**Table 4:** Percentage of mice developing hydrosalpinx.

Group	% mice with hydrosalpinx	Bilateral	Unilateral
PBS	100	66.7	33.3
Pgp3	100	83.3	16.7
CSPE/Pgp3	83.3	33.3	50
IL-12/Pgp3	83.3	33.3	50
CSPE/IL-12/Pgp3	50	0	50

### Biocompatibility assays

**Figure 9:**
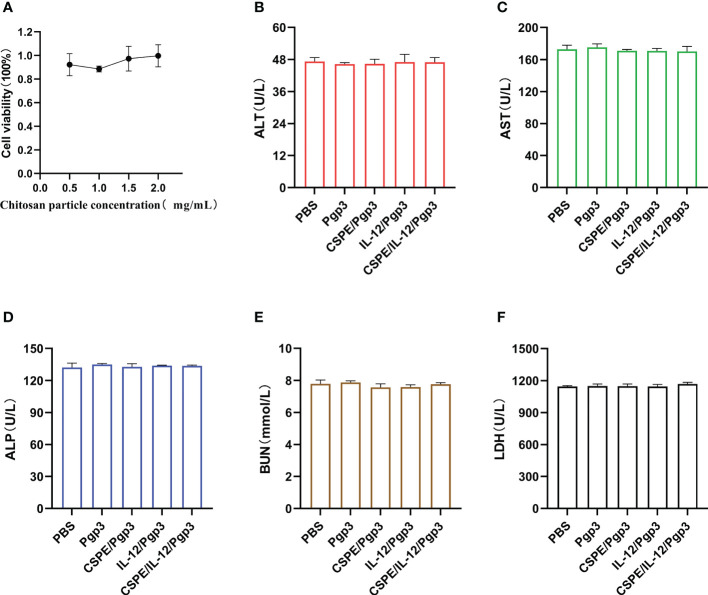
Biosafety evaluations. Macrophages were co-incubated with CSPE prepared through chitosan particles at different concentrations and liquid paraffin, and then CCK8 was used to test the macrophages viability. **(A)** Corresponding line chart of absorbance value (OD) represented cell viability. Serum samples were collected two weeks after the final immunization from immunized mice to detect ALT **(B)**, AST **(C)**, ALP **(D)**, BUN **(E)** and LDH **(F)** for immunopathology evaluation. The data are representative of three independent experiments, *P<0.05, **P<0.01, ***P<0.001.

The biosafety profile was assessed with regard to the macrophage toxicity effect caused by CSPE as well as the immunopathology in the serum. As seen from **Figure 9A**, after macrophages were cultured with CSPE prepared with different concentrations of chitosan particles and liquid paraffin for 24h, 90% cell viability was detected by CCK8. The results indicated limited toxicity effects on macrophages caused by CSPE. Furthermore, the levels of Alanine aminotransferase (ALT, **Figure 9B**), Aspartate aminotransferase (AST, **Figure 9C**), Alkaline phosphatase (ALP, **Figure 9D**), urea nitrogen (BUN, **Figure 9E**) and Lactate dehydrogenase (LDH, **Figure 9F**) in the serum of immunized groups were within the same range as those of the PBS group, suggesting no signs of adverse effects on the heart circulatory, liver, and kidney functions. Overall, there was acceptable biosafety with respect to CSPE and IL-12 as *C. trachomatis* vaccine adjuvant candidates.

## Discussion

*C. trachomatis* infections can be successfully treated depending on current therapy strategies, but limited benefits for protective immunity development and asymptomatic patients. Accordingly, vaccination of *C. trachomatis* is an extremely effective option in infection prevention and control, especially the implementation of safe and immunogenic recombinant subunit proteins. In this study, Pgp3 protein was applied to investigate protection in female mice against the *C. muridarum* genital challenge. Recent studies have confirmed that Pgp3 protein can maintain the homeostasis of host cells contributing to *C. trachomatis* intracellular survival including apoptosis resistance, autophagy induction, cells survival promotion, and immune evasion (53–56), which was essential for the pathogenesis of *C. trachomatis*. Importantly, Pgp3 was regarded as a promising antigen for subunit vaccine candidates due to existing immunogenicity (17). However, in addition to the robust secretion of Pgp3-specific antibodies, cellular immune responses are imperative to combat chlamydia infection. Under these circumstances, another major challenge in the vaccine development of *C. trachomatis* was failed to stimulate the cellular immune response partly because of the absence of an efficient and safe adjuvant platform. Thus, the combination of CSPE and IL-12 was applied to the adjuvant platform for Pgp3 vaccine, which may provide a vaccination strategy to achieve anti-chlamydia effectiveness.

Pickering emulsions were solely stabilized by microgel particles such as alum, cellulose, chitin, chitosan, starch, poly-(lactic-co-glycolic acid) (PLGA) nanoparticles, and so on (22–24). In contrast to the surfactant stabilized emulsion, Pickering emulsions eliminated the adverse effects including biological interaction or irritation caused by surfactants and blocked droplets coalescence due to oil/water interface absorbed hydrophobic solid particles, conferring enhanced safety and stability (24). The adjuvant functions of Pickering emulsions prepared by alum or PLGA were determined that enhanced the recruitment (22, 23), antigen uptake, and APCs activation to potently stimulate both humoral and cellular adaptive responses compared with conventional surfactant stabilized emulsions, indicating that Pickering emulsions may serve as a safe and efficient adjuvant platform for enhanced subunit vaccines. Ideally, the safety profile and accessible source of particle stabilizers were imperative for Pickering emulsions preparation. Asfour MH and other researchers have suggested that chitosan was suitable to stabilize oil/water Pickering emulsions owing to its polycationic properties and biosafety (25–28). Therefore, we prepared CSPE by homogeneous ultrasound under various circumstances to obtain the most optimized emulsion formulations for adjuvant application.

Actually, targeted distribution, immune recognition, uptake, processing, and presentation of antigens in the immune system were improved by small-size adjuvants, which can induce antigen migration to lymph nodes for activating APCs, T cells and B cells (57–59). However, transient retention of antigens at the injection site was caused by small-size adjuvants directly penetrating into the blood circulation, which was not beneficial for the formation of long-term immune memory (59, 60). Thus, rational regulation of droplet size was conducive to promoting antigens uptake and maintaining antigens residence time at the injection site. After optimizations, the CSPE with a suitable size was obtained (**Figures 1F**, **G**, **Table 1**). In addition, we revealed that chitosan tended to absorb the liquid paraffin/water interphase by rhodamine fluorescent sign (**Figure 2A**), indicating chitosan successfully stabilized oil/water interphase. Owing to the high specific surface area and positively charged chitosan surface (+33.2 ± 3mV, **Table 1**), CSPE may load proteins by electrostatic adsorption resulting in seldom adverse effects on proteins structure. As shown in **Figure 2B**, we confirmed that FITC-BSA was expectedly absorbed to CSPE interphase and speculated that the emulsion may load the antigen by electrostatic adsorption. Moreover, more than 90% of Pgp3 protein may be effectively absorbed by the optimized CSPE within 4h (**Figure 2C**). Furthermore, the efficient phagocytosis of CSPE/FITC-BSA by macrophages was detected by fluorescent staining (**Figure 3A**), indicating an enhancement of antigens uptake for beneficially cellular response achievement. Collectively, CSPE may be a hopeful adjuvant candidate for protein loading and delivery. Furthermore, the delivery systems not only promoted injected antigens residence but also increased the residence time of adjuvants. There is growing interest in developing a compound adjuvant system based on antigens delivery and immunopotentiator for vaccines. A promising area of investigation with relevant adjuvant applications stems from the knowledge on the immunostimulatory effect of IL-12 (29). On this basis, it is hypothesized that the residence time of IL-12 at the injection site may be prolonged through the use of delivery systems, which may be conducive to improvement of the immunomodulating effects of IL-12. To this end, a CSPE/IL-12 adjuvant system was applied for the Pgp3 protein vaccine to achieve robust immune responses.

Based on the immune-enhancing effects of IL-12 and the delivery potential of CSPE, an important finding was that all adjuvant immunized groups induced more Pgp3-specific IgG, IgG1, IgG2a, sIgA production and higher levels of IFN-γ, IL-2, TNF-α, IL-4 than Pgp3 immunized group in addition to IL-4 secretion of Pgp3/IL-12 immunized group. As demonstrated by high levels of IgG, IgG1, IgG2a, and sIgA, the humoral response generated by the CSPE/IL-12 adjuvant system was higher than CSPE and IL-12 adjuvants. Similarly, T-cell proliferation and TNF-α secretion in Pgp3/CSPE and Pgp3/IL-12 groups were significantly less than Pgp3/CSPE/IL-12 group. A substantial level of IFN-γ release was induced by CSPE adjuvant, while its IFN-γ level was less than induction by CSPE/IL-12. Therefore, neither individual adjuvant component was enough to induce a Th1 immune response, but CSPE/IL-12 was required to induce a stronger Th1 immune response. Intriguingly, CSPE was sufficient to induce a high level of IL-4, similar to that generated by CSPE/IL-12, which may be explained by no stimulation on IL-4 production and Th1 response biases of IL-12. The Th1 response biases of IL-12 were also reflected in higher IgG2a/IgG1 ratio while there was a balance between cellular and humoral immune response in Pgp3/CSPE/IL-12 groups in accordance with the IgG2a/IgG1 ratio. Collectively, we demonstrated that the CSPE/IL-12 adjuvant system for Pgp3 protein elicited strong cellular and humoral immune response. To test whether vaccine formulations would provide protection against chlamydial genital infection, in this regard, mice were intravaginally administrated with *C. muridarum*. Consistent with the robust immune response generated by Pgp3/CSPE/IL-12 vaccination, it was observed that significant decreases in the chlamydia organism shedding, the number of mice with positive vaginal cultures, hydrosalpinx and pathological changes, indicating significantly declining infection.

The Pgp3 protein efficacy of immune responses and *C. muridarum* prevention were greatly enhanced by CSPE/IL-12 adjuvant application, which may be explained by exploring the various stimulate pathways of the immune system by CSPE and IL-12. For instance, by increasing exposure of the protein vaccines to APCs, the immune cells may be activated by CSPE and respond by inducing chemokines release, which may result in the migration of immune cells into the injection site. The recruited immune cells may quickly endocytose the antigens and migrate to the lymph nodes (22, 23, 61). Another possible pathway is that antigens may migrate to the lymph node over a prolonged period of time because of the reservoir effect at the injection site. In addition, NK cells at the injection site were stimulated by IL-12 to produce IFN-γ to in turn stimulate APCs to take up antigens (33, 35), which may also be potentially prolonged by the Pickering emulsion depot effect. Therefore, further research will be needed to prove these conjectures for elucidating immune pathways. In addition, *C. trachomatis* serovars D~K (the genital tract biovar) may cause urogenital infected diseases, and serovars E is the most prevalent sexually transmitted chlamydia in humans (2, 62). In this study, the *C. muridarum* was used as a genital challenge model because the pathogenicity in genital tract infection of mice leaded by *C. muridarum* infection are highly similar with *C. trachomatis* infection (63, 64). However, this is still not 100% convincing that only depend on female mice model infected with *C. muridarum* when researchers studied adjuvants and vaccines. There are obviously important and promising that establish and improve the genital challenge model infected with *C. trachomatis* serovars E, which may be conducive to developing an effective human vaccine against *C. trachomatis*. While further research will need to elucidate the relative immune stimulating mechanism and improve the genital challenge model infected with *C. trachomatis*, the present data demonstrated that Pgp3/CSPE/IL-12 vaccination significantly accelerated the extinction of genital chlamydia and minimized the pathologic changes of hydrosalpinx occurrence.

## Conclusions

In summary, we confirmed a new adjuvants system strategy composed of CSPE and IL-12 for *C. trachomatis* subunit vaccine. CSPE was designed to provide an injection site storage for Pgp3 protein and the IL-12 and increase exposure of the Pgp3 protein to APCs. Combined vaccination of CSPE/IL-12 adjuvant system and Pgp3 protein elicited robust humoral and cellular immunity, which prevented chlamydia infection by eliminating the *C. muridarum* load and alleviating pathologic changes of fallopian tubes in mice. By exploiting an efficient and safe adjuvant strategy for subunit vaccine, this study may further propel the development of *C. trachomatis* vaccine to deal with challenge.

## Data availability statement

The original contributions presented in the study are included in the article/supplementary material. Further inquiries can be directed to the corresponding author.

## Ethics statement

The animal study was reviewed and approved by The Ethics Committee of the Animal Experiment Research Center at University of South China.

## Author contributions

MS and LZ conceived and designed the study, and the experiment was completed by MS, KS and LZ. MS and WL analyzed results and data. MS wrote this manuscript, YY and ZL revised this manuscript. All authors contributed to the article and approved the submitted version.

## Funding

This work was supported by the National Natural Science Foundation of China (No. 32070189 and 81772210), Hunan Provincial Natural Science Foundation of China (No.2021JJ30594), the Key Program of Hunan Provincial Department of Education (No.20A421), Clinical Research Project of University of South China (No.USCKF201902K01).

## Conflict of interest

The authors declare that the research was conducted in the absence of any commercial or financial relationships that could be construed as a potential conflict of interest.

## Publisher’s note

All claims expressed in this article are solely those of the authors and do not necessarily represent those of their affiliated organizations, or those of the publisher, the editors and the reviewers. Any product that may be evaluated in this article, or claim that may be made by its manufacturer, is not guaranteed or endorsed by the publisher.
